# Factors Associated with Quality of Life among Caregivers of People with Spinal Cord Injury

**DOI:** 10.1155/2021/9921710

**Published:** 2021-10-12

**Authors:** Ata Farajzadeh, Malahat Akbarfahimi, Saman Maroufizadeh, Negar Miri Lavasani

**Affiliations:** ^1^Department of Occupational Therapy, School of Rehabilitation Sciences, Iran University of Medical Sciences, Tehran, Iran; ^2^School of rehabilitation sciences, Ottawa university, Ottawa, Canada; ^3^Department of Occupational Therapy, School of Rehabilitation Sciences, Rehabilitation Research Center, Iran University of Medical Sciences, Tehran, Iran; ^4^School of Nursing and Midwifery, Guilan University of Medical Sciences, Rasht, Iran; ^5^School of Health and Social Development, Deakin University, Geelong, Australia

## Abstract

**Purpose:**

Often people with spinal cord injury (SCI) require help from their caregivers to carry out activities of daily living. Such assistance may affect caregiver quality of life (QoL). This study investigates the QoL and its associated risk factors among caregivers of people with SCI to find possible ways to increase their QoL. *Material and Method*. A convenience sample of 135 Iranian caregivers of people with SCI participated in a cross-sectional study from the Brain and Spinal Injury Repair Research Center of Tehran (BASIR), Iran, from June 2018 to October 2019. The World Health Organization's Quality of Life Questionnaire (WHOQoL-BREF), the Beck Depression Inventory-II (BDI-II), the Caregiver Burden Scale (CBS), and a demographic questionnaire were administered. Hierarchical multiple linear regression analysis was then applied to identify risk factors associated with caregiver QoL.

**Results:**

Moderate to highly significant negative correlations were observed between all domains of the WHOQoL scale and subscales of the CBS and the BDI-II. After controlling for demographic and clinical variables, depression, burden, and level of injury were found to predict caregiver QoL significantly. Furthermore, QoL was lower in caregivers of people with quadriplegia than paraplegia (*p* < 0.05).

**Conclusions:**

The level of injury, self-perceived caregiver burden, and depression are associated with QoL for the caregivers of people with SCI. A holistic approach incorporating caregiver training, psychological interventions, and adequate support may enable better QoL for these caregivers.

## 1. Introduction

Spinal cord injury (SCI) is a significant life event, with serious biopsychosocial consequences across multiple life domains for affected individuals, families, and friends [1, 2]. Improvements in medical and rehabilitation sciences have contributed to an increase in life expectancy for people with SCI [3]. As a result, these individuals may require sustained assistance in everyday occupations over many years [2, 4]. The family members of people with SCI, who become informal caregivers, are usually the primary source of assistance and facilitate activities such as eating, dressing, and toileting [5, 6]. In addition to assisting with personal care tasks, caregivers also assist with domestic and instrumental activities of daily living, such as shopping, homemaking, and financial management [7, 8].

Caregivers often transition to their new role with little or any prior preparation. Two factors that contribute to this are the rapid onset of SCI and the policies of healthcare systems in countries such as Iran that, on the one hand, rely heavily on informal caregivers post discharge, while, on the other hand, limiting hospital stays as short as possible to reduce costs. [6, 9]. Caregivers must cope with the demands of providing physical assistance and the care recipient's psychosocial needs, such as psychological symptoms and changes in social networks [10]. The new role creates additional psychosocial, physical, and financial impacts on caregivers, which are often referred to as caregiver burden [1, 4, 6]. Previous research has shown that many caregivers of people living with SCI experience depression and high levels of stress which affect their mental health and life satisfaction, with a subsequent negative impact on their QoL [1, 5, 11, 12]. QoL is a multidimensional concept and has been defined as an individual's perception of their position in life, within the context of their culture and value systems [9, 13]. The QoL of caregivers includes other factors related to their new role, such as caregivers' burden and family functioning toward performing caregiving jobs [14]. Caregiver QoL may also be impacted upon by the prioritisation of the care recipients' needs above their own, leading to a decline in a caregiver's life satisfaction in comparison with the general population [10].

While there are many studies on the QoL of people with SCI [15–17], there is a dearth of research on the QoL of their caregivers. Furthermore, caregiver studies tend to originate from developed countries [18, 19]. These papers mostly surveyed variables such as caregiver resilience, mental health, depression probability, and also the impact of aging in people with SCI on their caregivers [2, 5, 18, 20–22]. In addition, existing studies have mainly focused on psychological factors or are limited to a care recipient's and caregiver's demographic characteristics, as the main risk factors for poor QoL [1, 4, 22, 23]. For example, Wolf et al. (2014) examined relationships between psychological distress, health-related QoL, and burden, among 44 caregivers of people with SCI over 2 years, without assessing the predictive power of variables to enable healthcare team prioritize caregivers' needs. Although there is a high prevalence of SCI in developing countries [24], little research on caregiver burden has been conducted in these settings [9, 12]. Furthermore, the role of rehabilitation services including occupational therapy regarding the occupation of caregiving is not discussed adequately [24–26]. Historically, occupational therapists have focused on how individuals complete daily occupations. In fact, occupational therapists have an integral part in the rehabilitation phase of recovery and therefore have an obligation to provide support to the caregiver and care recipients to facilitate their reintegration into community. Fortunately, occupational therapists are well equipped to provide caregivers with the skills to be successful in a new occupation of caregiving. These skills include educating, training, and facilitating problem solving for a caregiver's concerns so that they are better able to care for themselves and the care recipient.

In recent years, the healthcare system in Iran has fundamentally changed, with significant emphasis on outpatient and home-based treatment settings. Such a change, by its very nature, has increased caregiver responsibilities [8, 12, 27]. This shift has also highlighted the role of occupational therapy to ameliorate the detrimental effects of caregiving on the carer's occupational balance and QoL [26].

In order to positively facilitate the caregiver role and maintain the occupational balance in caregivers' life, knowledge of the predictors of poor caregiver QoL is essential for an occupational therapist. Through such knowledge, occupational therapists can better target “at risk” caregivers and work to lessen the negative impacts of occupational imbalance as well as improve the occupational performance and social participation of caregivers [28]. Occupational therapists typically use a person-centered approach, with view to empowering individuals. Such a perspective also appreciates and incorporates appropriate cultural factors into an educational program. In fact, occupational therapists can empower caregivers in their new role by teaching them to use appropriate skills and solve problems efficiently [29]. To date, little emphasis in the literature has been placed on the caregivers of those living with SCI, with a preference given to conditions such as cerebral palsy or Alzheimer's [8, 29], and only general information is provided in the literature about SCI. Therefore, there appears to be an urgent need to support informal caregivers of those living with SCI, with the effect of easing caregiver burden and improving their QoL. Once caregiver burden and the risk factors that lead to poor QoL have been identified, occupational therapists can provide programs that are aimed at optimizing the quality of provided care and the QoL of both caregivers and care recipients [3, 30]. Therefore, this paper will address how much the caregivers' QoL can be influenced through the caregiving tasks. The specific aim was to investigate whether there is a correlation between QoL with carer burden and depression, and on the basis of these correlations, the purpose was also to predict the factors that can be recognised as the primary influences on QoL of caregivers. We proposed three main research questions that would be investigated in this paper. (1) Is there any correlation between the demographical characteristics of people with SCI and their caregivers and caregivers QoL? (2) Is there any correlation between the burden imposed on caregivers, the extent of caregiver depression, and QoL? (3) Which factors operate as the main determinant of poor QoL? According to Ziff et al., occupational therapists provide caregiver training to mitigate the risk of developing depression and burden for caregivers [31]. This study is aimed at detecting, addressing, and understanding factors predicting QoL of Iranian caregivers, to enable occupational therapists to facilitate the tailoring of a comprehensive educational program to mitigate the negative effects of caregiving in Iran.

## 2. Methods

This study used a cross-sectional, descriptive methodology. The local ethics committee of Iran University of Medical Sciences reviewed and approved the study design (93-D105-5537).

### 2.1. Recruitment and Study Population

A convenience sample of 135 Iranian caregivers of community dwelling people with SCI was recruited for this study. The inclusion criteria for caregivers were as follows: (a) a family member of the person living with SCI; (b) more than 11 hours a day spent in the role of caregiver; (c) at least six months of experience in the role of caregiver; (d) age range of 18-60; (e) ability to speak and understand Persian language; and (f) full literacy (reading and writing) in Persian. Caregivers with chronic conditions, such as diabetes, heart disease, or neurologic disorders, were excluded because chronic conditions can cause issues that might indirectly influence caregiving tasks; for example, their own condition may add to any depression or burden they are experiencing. Eligible participants were enrolled via the Brain and Spinal Injury Repair Research Center (BASIR) of Tehran, Iran, from June 2019 to October 2020. BASIR includes medical, psychological, and rehabilitation wards. Typically, SCI survivors (acute and chronic), from all parts of Iran, attend BASIR to receive a wide range of services. Services extend from acute inpatient spinal injury treatment through to transition back into the community. There is also an outpatient rehabilitation unit. Caregivers of people with SCI, who were attending the rehabilitation outpatient unit (occupational and physical therapy), were invited to participate in this study if they met the inclusion criteria. The included caregivers (*n* = 135) signed an informed consent document and completed the questionnaires in the presence of the principal investigator.

### 2.2. Data Collection

Demographic information about age, sex, education level, employment status, marital status, relationship to the individual with SCI, and level of injury of care recipients were initially gathered in a person-to-person survey. Carers were then asked to complete the Caregiver Burden Scale (CBS), Beck Depression Inventory-II (BDI-II), and World Health Organization's Quality of Life Questionnaire (WHOQoL-BREF) which are all available in Persian version.

### 2.3. Instrument

#### 2.3.1. The Caregiver Burden Scale (CBS)

The Caregiver Burden Scale (CBS) is a multidimensional scale assessing the subjective burden of care of people with chronic diseases [4, 32]. It includes 22 items and is divided into five domains: General Strain (eight items), Isolation (three items), Disappointment (five items), Emotional Involvement (three items), and Environment (three items). Each item is scored via a four-point Likert scale, ranging from one (not at all) to four (often). Total and subscale scores are obtained by summing the relevant items, with higher scores representing more significant perceived burden. The psychometric properties of the Persian version of the CBS have been established, including internal consistency, test-retest reliability, and construct validity [33]. In this study, Cronbach's alpha coefficient for the CBS domains ranged from 0.689 to 0.755 (except for environment subscale -0.559). This indicates that the set of items is closed and can be considered a group in each domain.

#### 2.3.2. Beck Depression Inventory-II (BDI-II)

The BDI-II is a 21-item self-report inventory designed to measure the intensity of depressive symptoms in psychiatric and nonpsychiatric adolescent and adult populations [34]. Items are scored on a four-point Likert scale ranging from zero (no symptoms) to three (severe symptoms). The total score can range from zero to 63, with higher scores indicating more significant depression levels. A total score of >13 indicates the presence of depressive symptoms. The Persian version of the BDI-II has shown satisfactory reliability and validity in Iranian populations [35]. Cronbach's alpha coefficient for the BDI-II in the present study was 0.870. This indicates that the set of items is closed and can be considered a group.

#### 2.3.3. World Health Organization's Quality of Life Questionnaire (WHOQoL-BREF)

The WHOQoL-BREF is one of the best-known tools for evaluating QoL and is available in more than forty languages including Persian [36]. The scale was developed in 1998 and assessed QoL over four domains: Physical Health (seven items), Psychological Health (six items), Social Relationships (three items), and Environmental Health (eight items). Scores for each domain can range from zero to 100, with higher scores indicating better QoL. The Persian version of WHOQoL-BREF has demonstrated adequate psychometric properties in the Iranian population [36]. In this study, Cronbach's alpha coefficient of the WHOQoL-BREF domains ranged from 0.624 to 0.737. This indicates that the set of items is closed and can be considered a group in each domain.

### 2.4. Data Analysis

Statistical analysis was carried out using IBM SPSS Statistics for Windows, version 16 (IBM Corp., Armonk, NY, USA). Descriptive analysis was undertaken initially, with continuous variables described using means and standard deviation (SD) and categorical variables described with frequencies and percentages. Pearson's correlation coefficient was performed to evaluate relationships between demographic characteristics, WHOQoL-BREF domains, CBS subscales, and the BDI-II. Differences between demographic characteristics and WHOQoL-BREF domains were also examined using an independent *t*-test and one-way ANOVA (followed by Duncan's test) as appropriate to discover which caregivers with specific demographic characteristics are most in danger of burden, depression, and experiencing a low level of QoL.

Hierarchical multiple linear regression analyses were then used to evaluate the relationships between the WHOQoL-BREF, CBS subscales, and BDI-II, controlling for caregiver demographic characteristics, e.g., age, gender, educational level, employment, and level of injury of people with SCI. The main purpose of multiple regressions is to predict the value of a dependent variable (QoL) based on several independent variables (burden, depression). Other purposes of regression analysis are to identify the strength of association (*R*^2^) between the dependent variable and several independent variables together and identify variables that significantly contribute to predicting the values of the dependent variable.

All models were checked for multicollinearity using tolerance and variance inflation factor (VIF). A tolerance < 0.1 and/or VIF > 5 indicates a multicollinearity problem, but none of the variables showed significant multicollinearity. Multicollinearity means that the independent variables in the regression equations are highly correlated.

## 3. Results

### 3.1. Caregiver Characteristics

Of the 135 caregivers included, 75 (55.0%) were female, 60 (45%) were male, 95 (70.4%) were married, 40 (30%) were single, 76 (56.3%) were unemployed, and 42 (31.1%) had tertiary (graduated from university) education. The mean age of caregivers was 36.85 ± 11.79 years (range: 18-60 years). One hundred and eleven affected individuals (82.2%) had a paraplegia, while 24 (17.8%) of them were living with quadriplegia. The relationship between people with SCI and their caregivers is as follows: parents (24%), spouse (23%), siblings (32%), children (15%), and others (6%).

### 3.2. Descriptive Statistics and Correlations among Study Variables

Table 1 presents the means, SDs, and intercorrelations across multiple variables. The mean BDI-II score was 15.38 ± 8.65 (range 0–48), with 69 caregivers (51.1%) exceeding the scale cut-off for depressive symptoms. WHOQoL-BREF domains showed significant but moderate negative correlations (*p* < 0.00) with the BDI-II and CBS subscales.

### 3.3. Relationship of QoL with Demographic Characteristics

The relationships between demographic characteristics of caregivers and the WHOQoL-BREF domains, using univariate analysis, are presented in Table 2. The greatest QoL was recorded in the Social Relationships domain, followed by the Psychological, Physical, and Environment domains. Married caregivers significantly scored lower than single caregivers on the Environment domain (*p* = 0.001) and parent caregivers scored significantly lower than others on all WHOQoL-BREF domains except Social Relationships. The caregivers of people with paraplegia recorded significantly higher QoL scores than caregivers for people with quadriplegia (*p* < 0.05). However, caregiver QoL was not significantly related to gender, educational level, or employment status.

### 3.4. Multiple Linear Regression Analysis

Hierarchical multiple linear regression analyses were performed to identify factors that were significantly independently related to the WHOQoL-BREF domains (Table 3).

#### 3.4.1. Physical Health Domain

According to the standardized regression coefficient in block 1, the injury level was significantly related to the Physical Health domain (*B* = 16.65, *p* < 0.001). Furthermore, parent caregivers scored higher than other caregivers on the Physical Health domain; the difference was not statistically significant (*B* = 10.54, *p* = 0.051). Model *R*^2^, when the demographic variables were in the Physical Health model, was equal to 0.192, suggesting that 19.2% of the variance in this domain was explained by the demographic variables. In block 2, among the CBS subscales, only the General Strain domain was negatively correlated with Physical Health (*B* = −1.88, *p* < 0.001). Moreover, depression was marginally related to Physical Health (*B* = −0.35, *p* = 0.052). When the CBS subscales were added to the model, there was a considerable improvement in the model (*R*^2^ = 52.5%, Δ*R*^2^ = 33.3, *R*^2^_adj_ = 47.4%, *F* = 10.30, *p* < 0.001). More specifically, an additional 33.3% of the variance in the Physical Health domain was explained by the CBS subscales and depression.

#### 3.4.2. Psychological Domain

In block 1, the demographic characteristics of caregivers explained 19.5% of the variance in the Psychological domain (*R*^2^ = 19.5, *R*^2^_adj_ = 15.0%, *F* = 4.38, *p* < 0.001). According to the standardized regression coefficient, only the level of injury was significantly related to the Psychological domain (*B* = 14.51, *p* < 0.001). In block 2, among the CBS subscales and depression, the Environment subscale of CBS and depression was negatively correlated with the Psychological domain (*B* = −1.63, *p* = 0.028; *B* = −0.67, *p* < 0.001, respectively). When the CBS subscales and depression were added to the model, there was a significant improvement in the model (*R*^2^ = 53.0%, Δ*R*^2^ = 33.6, *R*^2^_adj_ = 48.0%, *F* = 14.43, *p* < 0.001). More specifically, an additional 33.6% of the variance in the Psychological domain was explained by the CBS subscales and depression.

#### 3.4.3. Social Relationships Domain

Regarding the Social Relationships domain, in block 1, only the level of injury was significantly related to this domain (*B* = 11.17, *p* = 0.005). Model *R*^2^ in this step was 10.0%, suggesting that 10.0% of the variance in Social Relationships was explained by the level of injury. In block 2, depression was negatively related to Social Relationships (*B* = −0.84, *p* < 0.001). Among the CBS subscales, the Environment subscale of CBS was marginally correlated with Social Relationships (*B* = −1.59, *p* = 0.087). When the CBS subscales and depression were added to the model, there was a significant improvement in the Social Relationships model (*R*^2^ = 41.1%, Δ*R*^2^ = 23.9, *R*^2^_adj_ = 34.8%, *F* = 8.18, *p* < 0.001).

#### 3.4.4. Environment Domain

According to the standardized regression coefficients, the level of injury appeared to be the only variable in block one that was significantly related to the Environment domain (*B* = 8.49, *p* = 0.008). In block 2, among the CBS subscales, only the Environment subscale was significantly correlated with the Environment domain of the WHOQoL-BREF (*B* = −2.08, *p* = 0.007). When the CBS subscales and depression were added to the model, there was a significant improvement in the model (*R*^2^ = 40.0%, Δ*R*^2^ = 30.0, *R*^2^_adj_ = 33.6%, *F* = 10.09, *p* < 0.001). More specifically, an additional 23.0% of the variance in the environment domain was explained by the CBS subscales and depression.

## 4. Discussion

We found that QoL of caregivers of people with SCI was generally lower than reported for the general Iranian population [37], which is consistent with other studies based in Iran [9]. However, contrary to other studies in other countries, this paper showed no association between gender and the QoL of caregivers [13]. This study also differed from findings of other researchers regarding, caregivers' level of education and employment status. The finding of this study showed that education and employment status of the caregivers of patients with SCI were not associated with their QoL [9, 13, 38]. This could be due to the Iranian supportive culture and intimate emotional relationships between caregivers and care recipients, leading to both members of this relationship having a similar level of QoL. For that reason, caregivers are motivated to help with feelings of affection and obligation.

More than half of the caregivers reported symptoms of depression (51.1%), which indicate the stressfulness of caregiving tasks [39, 40]. In Rodakowski et al.'s study, 40 percent of caregivers of adults aging with SCI had significant depressive symptoms. Elliot et al. also reported that 50 percent of SCI caregivers, who were rated using the Center for Epidemiologic Studies Depression Scale (CES-D), had scores that exceeded the cut-off used to identify persons who may be at risk for depression. It is important to mention that CES-D has been sometimes criticised for its inability to distinguish distress from diagnosable depressive syndromes. However, the obtained figures have to be taken into account as an important subject due to a caregiver's depression can worsen the depression of SCI survivors and predict poor outcomes in rehabilitation and early discontinuation of care in the home [39].

As expected, there was a significant negative correlation between the patients' level of injury and the caregivers' QoL. This can be explained by the fact that SCI survivors with lower levels of independence require higher levels of care, resulting in physical exhaustion and interrupted sleep and eventually decreasing QoL for their caregivers. Researchers in two different studies found that a higher spinal level of impairment in people with SCI was associated with a greater burden on their caregivers [4, 5]. Importantly, it has been shown that occupational therapists can improve caregivers' QoL by assisting people with SCI to improve their independence in daily occupations through various methods, including environment modification or prescribing assistive technology, therefore decreasing the amount of imposed burden on caregivers [26].

The analysis of the findings of this study demonstrated a moderately to highly significant negative correlation between all domains of the CBS, the BDI-II, and the WHOQoL. The psychological domain of the WHOQoL had the highest negative significant correlation with the BDI-II scores, probably due to the experience of long-term stressors by caregivers, which impact on their QoL. This result is not surprising when considering developing countries where family members are frequently the only source of support for the person living with SCI [9]. Caregiving has previously been found to lead to a decrease in social engagement and to a lack of opportunities for positive emotions and experiences, all of which understandably impact on caregiver QoL [39]. Therefore, to enhance rehabilitation outcomes and achieve better results for SCI survivors, preventing depression in caregivers of people with SCI is highly recommended. Even though occupational therapists do not provide psychological interventions, understanding factors contributing to depression and caregiver burnout could enable occupational therapists to establish a comprehensive occupational profile for both caregiver and care recipients, to provide support for advocating for and producing well-designed occupational therapy caregiver training. These trainings might include environment adaptation, teaching transfer techniques, and introducing pain management procedures. For example, Corcoran and Gitlin in 2001 tried to clarify a caregiver's concerns of people with dementia by focusing on environmental intervention within the concept of home-based occupational therapy [29]. They declared that the first step to help caregivers in the process of caregiving is to find problems, analyse them, and find an environmental strategy. Consistent with these recommendations, this study tried to show that the first step for intervention is recognising the problems and the amount of burden which impact QoL.

The General Strain domain of the CBS and the physical domain of the WHOQoL demonstrated the highest negative significant correlation. These results are concordant with those studies that reported caregiving burden was negatively correlated with the caregivers' QoL [3, 4, 22]. In other words, as the caregiver burden increases, life satisfaction decreases and the caregiver has less control over their life, resulting in the manifestation of psychological issues [1, 4, 6, 13, 38, 41].

The healthcare system in Iran is consistent with developed countries, in terms of a shift to outpatient and home-based services [20]. However, in Iran, shifting from inpatient to outpatient rehabilitation places most of the burden on caregivers. Moreover, there are other determinants that might place an added burden on Iranian caregivers and make the process of caregiving more difficult. These include the lack of subsidisation for rehabilitation sessions by insurance companies, limited social support from governmental or nongovernmental agencies such as information services poor access to assistive technology (AT), and limited financial assistance required by families to access AT devices and other helpful services, such as practical home help services [12, 27]. Caregivers of people with SCI in developed countries identified information services, health professional services, practical support, and lifestyle services, as the main services required to assist them in the caregiving role [18]. So, the lack of information to train caregivers is the common problem in both Iran and developed countries. In other words, what also adds to caregiver burden is the lack of comprehensive caregiver training and education from healthcare professionals, including occupational therapists. We believe this is rooted in a lack of knowledge about caregivers' QoL predictors. Studies indicate that caregivers are dissatisfied with the lack of information resources and support available, mainly associated with the perception that family caregivers nowadays take responsibility for the care that previously was the domain of nursing and other health professionals [8, 42]. Another important reason for caregiver burden might be that healthcare professionals, especially occupational therapists, concentrate on the rehabilitation process of persons with SCI, because they do not have enough knowledge about the determinants of poor QoL of caregivers to tailor person-centered caregiving education. An occupational therapist should be very concerned about the presence of caregiver burden and stress, because it has the potential to hamper the long-term rehabilitation of persons with SCI and their caregivers.

Interestingly, the level of spinal cord injury appeared to be the only variable among demographic factors that was significantly related to the Environment domain, which means caregivers of people with a higher level of SCI suffer from more environmental difficulties. The correlation between both environmental domains of CBS and WHOQoL shows how environmental burden can influence caregivers' QoL. This correlation is consistent with basic assumptions in occupational therapy that considers the environment as one of the most important concepts in the occupational therapy evaluation and treatment process. In fact, occupational therapy researchers have long recognised the role of the environment as a treatment modality and have invented therapeutic models such as the Person-Environment-Occupation model (PEO) to show the role of each factor [43]. Caregivers are well positioned to instruct occupational therapists on specific cultural aspects and preferred ways of performing daily occupations, within their home and community environments. Such preferred ways incorporate values and beliefs important to the caregiver and their care recipient. Occupational therapists are wise to learn from such demonstrations and discussions and use this for effective interventions [29]. Occupational therapists can decrease caregiver burden by teaching caregivers how to appropriately modify the context and environment for particular occupations. Any training carried out by the occupational therapist must align with the cultural aspects of the particular context in which a caregiver provides care and should be tailored in a collaboration with caregivers, clients, and other therapists, to ensure a client-centered intervention.

Regression analysis demonstrated that depression, moderate to high caregiver burden, and the level of injury significantly predict QoL in caregivers. The level of injury is the only demographic variable that can be considered a predictor for QoL. In fact, the level of injury significantly correlated to the WHOQoL domains and predicted almost 20 percent of physical and psychological domains. The level of injury also predicted 10 and 17 percent of Social Relationships and Environment domains among all scattered variables. By assessing depression and burden, we discovered that roughly one-third of all domains of QoL, except for the environment, was predicted by BDI and CBS. This suggests that focusing on the burden and psychological state of caregivers may improve caregivers QoL by approximately 30 percent. Interestingly, 23 percent of the environment domain of QoL was predicted by CBS and BDI. Hence, the depression and domains of CBS would significantly predict probable QoL above and beyond caregivers' demographic characteristics. The Depression and General Strain domains of CBS have the highest predictive power of the physical domain of the WHOQoL, and depression and the environment of CBS have the highest predictive power of the other domains of the WHOQoL. Other studies in line with the present study showed that depression and caregivers' burden could predict the physical and mental health domains of the QoL of caregivers of people with Alzheimer's [44]. From our results, we can conclude that the risk factors most relevant to the QoL of the caregivers are the injury level of patients, caregiver burden, and depression.

In brief, the challenge for occupational therapy is how occupational therapists will tailor their interventions to impact both people with SCI and their caregivers effectively. To answer this question, they first should identify the issues that affect a caregiver's life. Finding such issues provides both challenges and opportunities for occupational therapists and leads to knowledge related to predictors for caregivers' QoL. By recognising QoL predictors, new ways of understanding and dealing with daily occupations of caregivers and their care recipients can be developed. This is radically important as SCI is a life-long and chronic condition. Caregivers may be required to provide support for the physical and psychological deficits resulting from SCI for a long time. Therefore, occupational therapists and other health professionals should determine the factors and then collaborate with the caregiver, to ameliorate their role as a caregiver. Although the current educational programs delivered to caregivers by occupational therapists are mostly individualised, having general knowledge about the essence of burden and its correlation with other psychological emotions such as depression can help therapists to individualize caregiver training, in order to mitigate risk factors for depression and caregiver burden. The therapist, in collaboration with the caregiver and the individual with SCI, can discuss risk factors of poor QoL and develop possible strategies that the caregiver can use to manage these factors. Such an individualised collaborative approach is in line with desirable client-centered practice. However, in the process of collaboration, it is important for the occupational therapist to remember that caregivers are persons who may require healthcare professional intervention for their own problems [8]. If the caregiver can be counted as the “hidden patient,” then more caregiver information must be obtained by special assessments, which can be conducted by an occupational therapist. The goal of caregiver assessments is to increase the health provider's awareness of caregiving demands and detect any concerns so that interventions can be tailored to prevent adverse outcomes and to grant a better perception of the related predictors. Occupational therapists who have the knowledge to enable caregivers to feel confident in their ability to provide care, identify their own needs, and maintain a balance between their own life and caregiving role would be very successful in their therapy [31]. Occupational therapists with the knowledge of predictors in QoL and expertise in occupation and analysing role demands, patient function, and their needs are in the best position to tailor a most suitable educational package for caregivers.

### 4.1. Limitations

This study has several limitations that should be noted. Firstly, this was a single-center study with relatively small sample size. Secondly, a convenience sampling method was used to collect the data. Such sampling limits the generalisability of the findings. Thirdly, not all factors that affect QoL were included in this study. In addition, we excluded caregivers with chronic disease, to decrease the probability of potential interference from the burden created by their own condition with the burden resulting from their caring tasks. Further studies, particularly longitudinal studies with a larger number of caregivers, are required to disentangle the nature of the relationship between QoL and its determinant factors.

## 5. Conclusion

Caregiving is an essential role, and occupational therapists should be aware of how to address and develop effective caregiving training in order to decrease the amount of burden and depression experienced by caregivers. In order to assist caregivers in having a better QoL, the healthcare team, especially occupational therapists, need to better understand the possible QoL issues for caregivers and the factors impacting them. This study is aimed at investigating QoL and associated demographic risk factors for caregivers of people with SCI. The predictive power of depression and caregiver burden in this study implies that interventions should focus on empowering family caregivers through providing proper training, supporting caregivers, and facilitating access to the necessary resources for managing the caregiver burden imposed on caregivers, especially caregivers of people with a higher level of SCI. Future studies will address interventions to deal with QoL predictors.

## Figures and Tables

**Table 1:** Means, standard deviations, and correlations among study variables (*n* = 135).

		1	2	3	4	5	6	7	8	9	10
1	Depression	1									
2	General Strain-CBS	0.659	1								
3	Isolation-CBS	0.520	0.697	1							
4	Disappointment-CBS	0.575	0.705	0.544	1						
5	Emotional Involvement-CBS	0.547	0.601	0.549	0.497	1					
6	Environment-CBS	0.439	0.574	0.502	0.458	0.557	1				
7	Physical Health-QoL	-0.567	-0.691	-0.468	-0.541	-0.447	-0.456	1			
8	Psychological-QoL	-0.639	-0.601	-0.417	-0.549	-0.450	-0.476	0.705	1		
9	Social Relationships-QoL	-0.576	-0.506	-0.404	-0.432	-0.419	-0.409	0.648	0.673	1	
10	Environment-QoL	-0.447	-0.479	-0.366	-0.450	-0.400	-0.422	0.581	0.615	0.654	1
	Possible Range	0-63	8-24	3-12	5-20	3-12	3-12	7-35	6-30	3-15	8-40
	Observed Range	0-48	9-32	3-12	6-20	3-11	3-12	9-32	8-28	4-15	10-38
	Mean	15.38	20.20	6.27	12.84	5.72	7.38	55.93	56.02	60.18	50.62
	SD	8.65	4.53	2.11	2.88	1.76	1.86	17.01	15.67	17.40	14.55

SD: standard deviation; CBS: Caregiver Burden Scale; QoL: quality of life. All *p* < 0.001.

**Table 2:** Relationship of WHOQOL-BREF domains with demographic characteristics in caregivers of people with SCI using univariate analysis.

	Physical Health		Psychological		Social Relationships		Environment	
	Mean ± SD	*P*	Mean ± SD	*P*	Mean ± SD	*P*	Mean ± SD	*P*
Sex		0.585		0.262		0.835		0.929
Male	56.75 ± 15.64		57.55 ± 15.59		60.18 ± 17.60		51.14 ± 14.44	
Female	55.13 ± 18.10		54.49 ± 15.71		60.19 ± 17.35		50.11 ± 14.69	
Marital status		0.378		0.255		0.687		0.001
Single	57.85 ± 17.33		58.22 ± 12.14		60.76 ± 17.58		58.05 ± 13.45	
Married	55.01 ± 16.90		54.85 ± 16.90		60.07 ± 17.40		48.61 ± 14.11	
Education level		0.933		0.255		0.340		0.751
Nonacademic	55.94 ± 15.29		54.82 ± 14.76		60.79 ± 15.80		51.14 ± 12.08	
Academic	55.67 ± 20.51		58.14 ± 17.50		58.33 ± 20.54		52.00 ± 19.02	
Employment		0.394		0.104		0.215		0.173
Unemployed	54.45 ± 16.81		53.40 ± 15.69		58.38 ± 18.98		50.50 ± 13.81	
Employed	56.97 ± 17.20		57.81 ± 15.48		62.13 ± 15.95		52.93 ± 14.99	
Relationship status		0.044		0.018		0.154		0.001
Parents	48.69 ± 17.63^b^		48.84 ± 16.30^b^		58.81 ± 16.92^a^		47.69 ± 11.72^a^	
Spouse	55.48 ± 13.26^a,b^		56.10 ± 15.42^a,b^		61.29 ± 17.62^a^		51.16 ± 12.34^b,c^	
Brother/sister	57.42 ± 19.14^a,b^		56.53 ± 16.91^a,b^		64.33 ± 18.25^a^		56.37 ± 15.84^a,b,c^	
Son/daughter	62.10 ± 15.31^a^		63.10 ± 10.30^a^		70.29 ± 15.40^a^		63.19 ± 12.19^a^	
Other	61.12 ± 11.92^a^		60.25 ± 8.91^a^		68.00 ± 15.19^a^		60.25 ± 17.39^a,b^	
Level of injury		<0.001		<0.001		0.004		0.016
Paraplegia	58.77 ± 15.90		58.51 ± 14.17		61.70 ± 16.36		51.89 ± 14.13	
Tetraplegia	42.33 ± 15.64		43.54 ± 16.67		51.17 ± 19.32		45.54 ± 14.65	

SD: standard deviation. ^a–c^Groups followed by the same letter are not significantly different at the 0.05 level.

**Table 3:** Results of hierarchical multiple linear regressions, including factors related to WHOQOL-BREF domains in caregivers of people with SCI.

	Physical Health		Psychological		Social Relationships		Environment	
	*B* (SE)	*p*	*B* (SE)	*p*	*B* (SE)	*p*	*B* (SE)	*p*
*Block 1: demographics*								
Age	0.13 (0.21)	0.523	-0.04 (0.19)	0.829	-0.13 (0.23)	0.578	0.02 (0.18)	0.893
Sex (male = 0)	-2.48 (3.04)	0.415	-3.55 (2.80)	0.207	-0.79 (3.28)	0.809	-0.49 (2.63)	0.853
Marital status (single = 0)	-3.34 (3.58)	0.352	-1.52 (3.29)	0.645	0.21 (3.86)	0.956	-9.12 (3.10)	0.004
Education level (secondary = 0)	-2.28 (3.02)	0.352	0.84 (2.78)	0.762	-5.10 (3.26)	0.120	-1.98 (2.61)	0.450
Employment (single = 0)	-1.88 (3.29)	0.569	-0.41 (3.03)	0.893	2.27 (3.56)	0.524	-1.33 (2.85)	0.642
Relationship (parents = 0)	10.54 (5.35)	0.051	6.05 (4.92)	0.221	1.89 (5.77)	0.744	6.50 (4.63)	0.162
Level of injury (paraplegia = 0)	-16.65 (3.63)	<0.001	-14.51 (3.34)	<0.001	-11.17 (3.92)	0.005	-8.49 (3.14)	0.008
Model characteristics	*R* ^2^ = 19.2%, *R*^2^_adj_ = 14.8%, *F* = 4.31, *p* < 0.001		*R* ^2^ = 19.5%, *R*^2^_adj_ = 15.0%, *F* = 4.38, *p* < 0.001		*R* ^2^ = 10.0%, *R*^2^_adj_ = 5.1%, *F* = 2.02, *p* = 0.057		*R* ^2^ = 17.2%, *R*^2^_adj_ = 12.7%, *F* = 3.77, *p* = 0.001	
*Block 2: CBS subscales and BDI-II*								
General Strain-CBS	-1.88 (0.44)	<0.001	-0.63 (0.40)	0.117	-0.37 (0.50)	0.470	-0.22 (0.42)	0.597
Isolation-CBS	0.45 (0.76)	0.550	0.57 (0.70)	0.416	0.15 (0.87)	0.861	0.22 (0.72)	0.761
Disappointment-CBS	-0.25 (0.55)	0.647	-0.59 (0.51)	0.246	-0.25 (0.63)	0.688	-0.81 (0.52)	0.127
Emotional Involvement-CBS	0.01 (0.86)	0.990	-0.19 (0.79)	0.814	-0.49 (0.99)	0.622	-0.82 (0.82)	0.316
Environment-CBS	-0.073 (0.80)	0.366	-1.63 (0.73)	0.028	-1.59 (0.92)	0.087	-2.08 (0.76)	0.007
BDI-II	-0.35 (0.18)	0.052	-0.67 (0.16)	<0.001	-0.84 (0.20)	<0.001	-0.24 (0.17)	0.158
Model characteristics	*R* ^2^ = 52.5%, Δ*R*^2^ = 33.3%, *R*^2^_adj_ = 47.4%, *F* = 10.30, *p* < 0.001		*R* ^2^ = 53.0%, Δ*R*^2^ = 33.6%, *R*^2^_adj_ = 48.0%, *F* = 14.43, *p* < 0.001		*R* ^2^ = 40.0%, Δ*R*^2^ = 30.0%, *R*^2^_adj_ = 33.6%, *F* = 10.09, *p* < 0.001		*R* ^2^ = 41.1%, Δ*R*^2^ = 23.9%, *R*^2^_adj_ = 34.8%, *F* = 8.18, *p* < 0.001	

BDI-II: Beck Depression Inventory-II; CBS: Caregiver Burden Scale; *B*: unstandardized coefficient; SE: standard error.

## Data Availability

The research data used to support the findings of this study are included within the article.

## Additional Points

*Implication for Rehabilitation*. (1) Spinal cord injury leads to depression, high levels of stress, and diminished quality of life due to the high physical, emotional, and social burdens in caregivers. (2) Identifying the caregivers' QoL determinants leads to improvement in the quality of caregiving tasks and the QoL of both caregiver and care recipient. (3) Occupational therapists, by identifying risk factors of QoL for caregivers of people with SCI, can tailor more appropriate interventions to achieve improved occupational balance between the competing occupations of caregivers. (4) It is an essential issue for occupational therapists to bear in mind that the condition of care recipient and the caregiver are tied to each other, so an intervention that addresses both SCI survivors and their caregivers will be more beneficial.
